# Intravascular emboli relates to immunosuppressive tumor microenvironment and predicts prognosis in stage III colorectal cancer

**DOI:** 10.18632/aging.203451

**Published:** 2021-08-26

**Authors:** Xiangping Song, Di Xie, Fengbo Tan, Yuan Zhou, Yuqiang Li, Zhongyi Zhou, Qian Pei, Haiping Pei

**Affiliations:** 1 Department of General Surgery, Xiangya Hospital, Central South University, Changsha, P.R. China; 2 Department of Geriatric Surgery, Xiangya Hospital, Central South University, Changsha, P.R. China; 3 Department of Neurology, The Third Xiangya Hospital, Central South University, Changsha, P.R. China; 4 The National Clinical Research Center for Geriatric Disorders, Xiangya Hospital, Central South University, Changsha, P.R. China

**Keywords:** intravascular emboli, colorectal cancer, tumor infiltrating lymphocyte, tumor microenvironment, prognosis

## Abstract

Background: Stage III colorectal cancer (CRC) patients experience varying degrees of prognosis even if receiving standard therapeutic regimes. Intravascular emboli (IVE), a type of vascular invasion, impacts the clinical outcome in CRC. In this study, we confirmed the role of IVE in predicting the prognosis of stage III CRC patients and characterized the tumor microenvironment (TME) of CRC with IVE.

Methods: Data from 220 consecutive patients (cohort 1) with stage III CRC undergoing radical surgery was collected retrospectively between January 2009 to December 2014. According to the presence of IVE, which was confirmed by two independent pathologists, patients were classified into two groups. Univariate and multivariate Cox regression analyses were performed to evaluate the relation of IVE presence to patients’ prognosis. The association between IVE and clinicopathological factors was also analyzed. Furthermore, differentially expressed genes (DEGs) and gene set enrichment analyses (GSEA) were performed to describe features of the TME based on microarray data consisting of 6 patients. Tumor tissues from a separate cohort of 73 patients with stage III CRC (cohort 2) collected between June 2014 and December 2015 were used to analyze tumor-infiltrating lymphocyte (TIL) by immunohistochemistry (IHC) staining.

Results: IVE was observed in 126 (57.3%) patients and could serve as an unfavorable independent prognostic predictor (P < 0.001) as well as lymph node metastasis (P < 0.05) and tumor location (P < 0.05). Additionally, patients with IVE had a higher neutrophil percentage (P = 0.002) and lower lymphocyte percentage (P = 0.002) relative to those without IVE. CRC with IVE had a significantly different profile of DEGs compared to CRC without IVE, and GSEA showed chronic inflammatory and immunosuppressive TME may promote IVE development. In cohort 2, tumors with IVE had fewer CD3^+^ TILs in the stromal region, as well as fewer CD8^+^ TILs in both stromal and tumoral regions relative to those without IVE.

Conclusion: IVE, which was related closely to a chronic inflammatory and immunosuppressive TME, forecasted a worse prognosis of stage III CRC patients and may be taken into consideration when a therapeutic strategy is decided upon.

## INTRODUCTION

Cancer is a major threat to human life expectancy among noncommunicable diseases worldwide. According to the GLOBOCAN 2018 estimates of cancer incidence and mortality, colorectal cancer ranks fourth and second for incidence and mortality, respectively [1].

Hematogenous spread, or vascular invasion, of CRC is the major way to develop distant metastasis which may ultimately result in death. Vascular invasion can be classified into three types: tumor cells outside of the veins, tumor cell emboli in the lumen of veins, tumor cell destruction of vein walls [2]. The IVE was defined as a rounded mass of tumor in an endothelium-lined space either surrounded by a rim of smooth muscle or containing red blood cells referring to the second type of vascular invasion [3–5]. The invasion-metastasis cascade can be described briefly as follow: tumor cells exit their primary growth site and enter the vessels, survive in the circulation and arrest at a distant organ, followed by adaptation and survival in the foreign microenvironment of distant tissues [6]. Circulating tumor cells (CTCs) or circulating tumor-derived DNA (ctDNA) can be used to detect minimal residual disease (MRD) after surgery, monitor treatment response and predict prognosis, however, several limitations still remain [7–10].

IVE generally escapes from immune surveillance and will develop distant metastasis finally [2, 4, 5, 11, 12], but the mechanism underlying IVE formation is unclear. The tumor microenvironment plays an important role in cancer development, metastasis and drug resistance [13] and may also play a role in IVE progress. The tumor-node-metastasis (TNM) stage system has contributed to a great extent to directing the management and treatment of CRC. With the development of precise treatment and insights into CRC, many more factors should be taken into consideration to optimize decision-making [14].

Patients who are diagnosed with early-stage CRC and receive curative treatments have a better prognosis. The five-year survival rate is 90% for patients with localized stages of CRC (there is no sign that cancer has spread outside of the colon or rectum). For patients with regional stage CRC (cancer has spread outside the colon or rectum to nearby structures or lymph nodes), the five year survival rate is 71%, which is an improvement from previous decades [15]. Though patients with stage III CRC often receive radio-/chemotherapy systematically along with surgery, the clinical prognosis of stage III CRC varies a lot and metastasis is the leading cause of death. Previous studies suggest patients with IVE have a worse prognosis including CRC [2–5, 11, 12]. CRC patients with IVE should be treated as another subtype.

In our study, we evaluated the prognostic significance of IVE in patients with stage III CRC after radical surgery and profiled the DEGs and tumor microenvironment characteristics of CRC with IVE.

## MATERIALS AND METHODS

### Patients and clinical follow-up

Data from consecutive patients with CRC were collected in Xiangya Hospital, Hunan, China between January 2009 to December 2014. The inclusion criteria required that patients had received radical surgery (R0 excision), confirmed stage III CRC histologically according to the AJCC7th classification. The exclusion criteria included: anti-tumor therapy before surgery; colorectal cancer with intestinal perforation; vascular disorder or inflammation-related diseases; and incomplete clinicopathological data. Finally, 220 patients were included in cohort 1. The last follow-up time was December 2017. Disease-free survival (DFS) was defined as the interval from radical surgery to recurrence or metastasis, the appearance of secondary colon or rectal cancer, or death, whichever occurred first. Overall survival (OS) was defined as the interval from radical surgery to mortality, or it was censored at the last known date alive. Similarly, 73 patients diagnosed with stage III CRC between June 2014 and December 2015 in the same institution were included in cohort 2.

For microarray analysis, six patients diagnosed with stage III CRC post-surgery were selected. Both cancer tissue and paired adjacent non-cancerous tissue were collected and stored in liquid nitrogen. Three patients were presenting with IVE and the other three were not. Four groups were defined as follows: cancer tissues with IVE (IVE group) and paired adjacent non-cancerous tissue (group 1); cancer tissues without IVE (non-IVE group) and paired adjacent non-cancerous tissue (group 2).

### Clinicopathological data

Blood laboratory measurements were carried out within 7 days before surgery. All patient-related data were retrieved from the medical record database, including blood count, comprehensive metabolic test, coagulation related indices, tumor markers including carcinoembryonic antigen (CEA), carbohydrate antigen 199 (CA199) and CA242, and demographic information and postoperative pathological results. The diagnostic criteria for IVE were described in detail previously [3]. Briefly, there was a cluster of cancer cells in blood vessel confirmed by Hematoxylin and Eosin (H&E) or immunohistochemistry (IHC) staining [3]. A minimum of 4 paraffin tumor blocks were used to optimize detection. For each block, we examined the entire filed to make sure weather there is IVE presence or not. When we encountered difficulties in identification and distinguishing sample features from lymphatic vessel invasion (LVI) in hematoxylin and eosin (H&E) slices, we marked the blood vessel endothelium and lymphatic vessel endothelium, with CD34 and D2-40 IHC staining, respectively (Supplementary Figure 1). The presence of IVE was confirmed by two independent pathologists.

### Immunohistochemistry staining and TILs assessment

Immunohistochemistry staining was performed as previously described [3, 16]. Briefly, the sections were dewaxed and rehydrated. Then, the sections were immersed in 10 mmol/L sodium citrate buffer (pH = 6), boiled and allowed to cool for 20 minutes to repair the sealed antigen. The sections were then incubated in 3% hydrogen peroxide solution for 25 minutes to block endogenous catalase. After blocking with 3% BSA, the sections were incubated with mouse monoclonal antibody against human CD8 (1:400 dilution, ab199016, Abcam) or rabbit polyclonal antibody against human CD3 (1:200 dilution, gb11137, Servicebio) overnight at 4° C, followed by incubation with goat anti-rabbit (1:200 dilution, GB23303, Servicebio) or anti-mouse (1:200 dilution, GB23301, Servicebio) IgG H&L (HRP) antibody (1:200 dilution, ab205718, Abcam) for 50 minutes at room temperature. The sections were stained with 0.05% diaminobenzidine tetrahydrochloride (DAB) for 5 minutes and then counterstained with hematoxylin, dehydrated, and mounted. The whole slide image was scanned by an automatic digital slide scan system (KF-PRO-400, KFBIO) and viewed through K-Viewer software (KFBIO). For positive cells counting, three fields (20×) of each region (stomal, tumoral, and boundary region in the tumor area) were selected randomly and counted blindly by covering IVE status.

### RNA isolation and gene expression profiling

The Agilent Human Gene Expression (8*60K, Design ID: 039494) was used in this experiment. Total RNA was isolated from 12 frozen samples and quantified by NanoDrop ND-2000 (Thermo Scientific). RNA integrity was assessed using Agilent Bioanalyzer 2100 (Agilent Technologies). The sample labeling, microarray hybridization, and washing were performed based on the manufacturer’s standard protocols. Briefly, total RNA was transcribed to double-stranded cDNA, then synthesized into cRNA and labeled with Cyanine-3-CTP. The labeled cRNAs were hybridized onto the microarray plate. After washing, the arrays were scanned by the Agilent Scanner G2505C (Agilent Technologies).

Feature Extraction software (version10.7.1.1, Agilent Technologies) was used to analyze array images to get raw data. Genespring (version13.1, Agilent Technologies) was employed for basic analysis of the raw data. To begin with, the raw data was normalized using the quantile algorithm. Probes for which 100% of the values in any of all available groups flagged as "Detected" were chosen for further data analysis. The number of differentially expressed genes (DEGs) were refined via a fold change as well as *P* value (*t*-test) cutoff. Hierarchical clustering was performed to display the distinguishable genes’ expression patterns among samples. All the analysis was performed on the R studio platform using packages including limma, gplots, and ggplot2.

Gene set enrichment analysis [17] was performed on GSEA software 3.0 to verify the biologic phenotype between groups. All prior gene sets were downloaded from MsigDB (Molecular Signatures Database).

The study was approved by the Ethics Review Committee of Xiangya Hospital, Central South University and performed in accordance with the Declaration of Helsinki.

### Statistics

Association between IVE and clinical characteristics was assessed by Chi-square (χ2) test. The *t*-test was used to analyze the differences between the means of two groups. Data are presented as mean ± s.d. Univariate and multivariate Cox regression analyses were performed to evaluate clinical characteristics related to DFS and OS. Survival curves were made using the Kaplan–Meier method using GraphPad Prism 7.0 and compared by the log-rank test. All statistical analyses were performed using the Statistical Package for Social Sciences version 25.0 (SPSS Inc., Chicago, IL, USA). A two-sided *P* < 0.05 was considered statistically significant. In GSEA, enrichment results satisfying a nominal *P*-value cutoff of 0.05 with a false discovery rate (FDR) q-value < 0.25 were considered statistically significant.

## RESULTS

### Patient characteristics

Cohort 1 included 220 patients with stage III colorectal cancer. All the patients received radical surgery at the Department of Gastrointestinal Surgery of our hospital from January 2009 to December 2014. The mean age and age range at the time of diagnosis were 69 years old and 14 to 83 years old, respectively. The ratio of male to female was 126/94. Among all patients, 84 (38.2%) had colon cancer and 136 (61.8%) had rectal cancer. There were 161 patients received adjuvant chemotherapy after surgery, among which there were only 8 receiving radiotherapy at the same time, 136 and 17 getting FOLFOX4/6 and XELOX, respectively. Laboratory results from pre-operation blood tests, including protein levels, hepatorenal function exam, serum electrolytes, various blood cell counts and coagulation-related indices, are showed in Supplementary Table 1. The last date of follow-up was December 2017 and the median follow-up duration was 64 months (range from 15 to 106 months). Cohort 2 included 73 patients, of which there were 30 patients with IVE.

### Survival analysis

**Figure 1:**
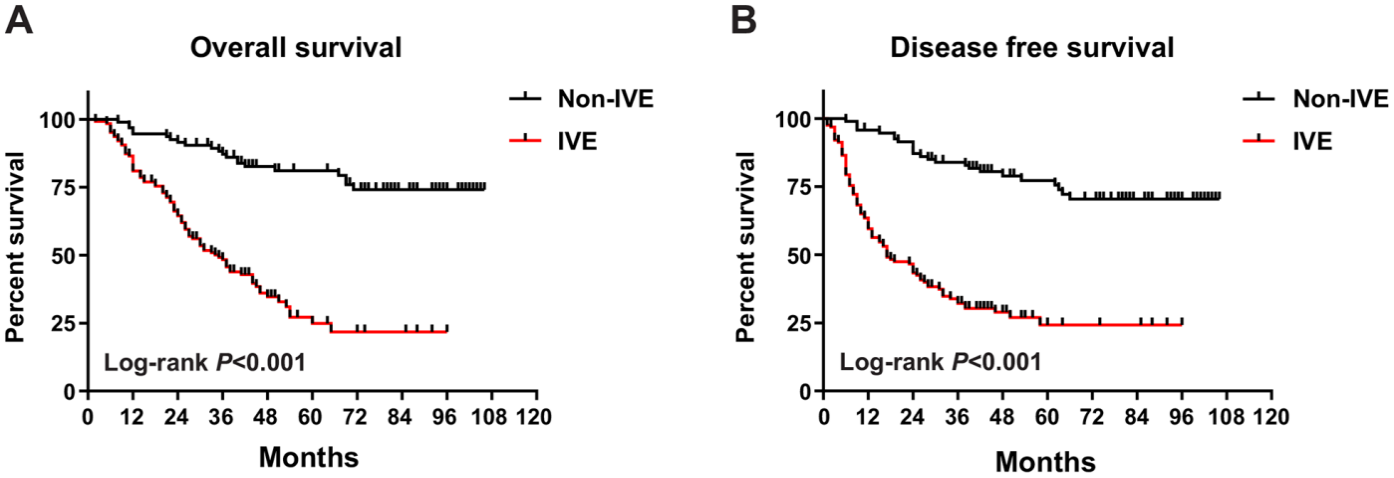
**OS and DFS in IVE and non-IVE CRC patients.** (**A**) IVE patients had worse OS compared with non-IVE patients; (**B**) IVE patients had worse DFS compared with non-IVE patients.

To assess the prognostic significance of some clinicopathological characteristics and IVE in patients with stage III CRC in cohort 1, univariate and multivariate analyses were applied. In univariate analysis, the results revealed that IVE, lymph node metastasis, tumor location, histologic type, obstruction, and serum CA199 levels were associated with both OS and DFS (*P* < 0.05). On the other hand, gross tumor morphology was only related to OS (*P* = 0.026) but not DFS (*P* = 0.078) (Table 1). All factors mentioned above, as well as the tumor invasion depth (*P* < 0.1), were included in the multivariate analysis, which suggests IVE was statistically significant for both OS (HR: 4.486, 95%CI: 2.638-7.631; *P* < 0.001) and DFS (HR: 4.949, 95%CI: 2.996-8.174; *P* < 0.001), as well as lymph node metastasis and tumor location (Table 2). Of the 220 patients, 113 developed tumor recurrence (non-IVE group, 24 versus IVE group, 89) and 103 had cancer-related deaths (non-IVE group, 21 versus IVE group, 82). Kaplan-Meier analysis and log-rank tests were applied to assess for differences in OS and DFS between groups characterized by IVE presence or not. It showed patients with IVE had a worse outcome after surgery and median OS and DFS of stage III CRC patients with IVE were 35 and 17 months, respectively. (Figure 1A, 1B).

**Table 1:** Univariate analysis in relation to OS and DFS.

**Parameters**		**OS**				**DFS**		
		***P*-value**	**HR**	**95%CI**		***P*-value**	**HR**	**95%CI**
Age	< 60 years							
	≥ 60 years	0.418	1.176	0.795-1.739		0.887	1.028	0.705-1.498
Gender	Male							
	Female	0.322	0.819	0.552-1.215		0.460	0.868	0.596-1.264
Tumor location	Left colon	**0.020**				**0.007**		
	Right colon	0.636	1.189	0.580-2.437		0.801	1.093	0.546-2.190
	Rectum	0.018	2.001	1.128-3.550		0.012	2.022	1.164-3.512
Histologic type	Well/moderate	**<0.001**				**0.007**		
	Poor	0.000	2.699	1.747-4.169		0.002	1.975	1.292-3.018
	Mucinous/mix	0.146	1.546	0.860-2.779		0.648	1.144	0.643-2.035
Tumor morphology	Ulcerative	**0.026**				0.078		
	Infiltrating	0.032	2.184	1.068-4.464		0.089	1.851	0.911-3.758
	Protruded	0.318	0.809	0.533-1.277		0.325	0.82	0.552-1.218
Tumor size	< 5 cm							
	≥ 5 cm	0.501	0.872	0.584-1.301		0.389	0.846	0.577-1.239
Obstruction symptoms	NO							
	YES	**0.002**	2.839	1.469-5.487		**0.001**	2.868	1.530-5.379
Invasive depth	T1-2							
	T3-4	0.064	2.178	0.954-4.969		0.060	2.084	0.970-4.480
Lymph node metastasis	pN1							
	pN2	**<0.001**	3.78	2.497-5723		**<0.001**	2.883	1.962-4.238
CEA	< 5 ng/ml							
	≥ 5 ng/ml	0.332	1.229	0.810-1.865		0.628	1.105	0.737-1.658
CA199	< 35 kU/L							
	≥ 35 kU/L	**0.028**	1.672	1.056-2.647		**0.039**	1.602	1.025-2.505
CA242	< 20 kU/L							
	≥ 20 kU/L	0.313	1.296	0.783-2.143		0.537	1.17	0.711-1.925
IVE	NO							
	YES	**<0.001**	5.224	3.188-8.561		**<0.001**	5.161	3.250-8.198

Abbreviations: CEA, carcinoembryonic antigen; CA199, carbohydrate antigen 199; CA242, carbohydrate antigen 242; IVE, intravascular emboli.

**Table 2:** Multivariate analysis in relation to OS and DFS.

**Parameters**		**OS**				**DFS**		
		***P*-value**	**HR**	**95%CI**		***P*-value**	**HR**	**95%CI**
Tumor location	Left colon	0.112				**0.015**		
	Right colon	0.548	1.272	0.580-2.789		0.497	1.303	0.606-2.801
	Rectum	**0.050**	1.938	0.999-3.760		**0.009**	2.337	1.239-4.408
Histologic type	Well/moderate	0.646				0.648		
	Poor	0.512	1.188	0.710-1.987		0.354	0.791	0.481-1.300
	Mucinous	0.413	1.318	0.681-2.551		0.910	0.964	0.506-1.835
Tumor morphology	Ulcerative	0.305				0.401		
	Infiltrating	0.194	1.679	0.769-3.666		0.253	1.566	0.726-3.379
	Protruded	0.532	0.86	0.535-1.381		0.589	0.887	0.575-1.369
Obstruction	NO							
	YES	0.547	0.125	0.599-2.631		0.281	1.464	0.733-2.925
Invasive depth	T1-2							
	T3-4	0.456	1.381	0.591-3.227		0.325	1.481	0.677-3.237
Lymph node metastasis	N1							
	N2	**0.001**	2.334	1.445-3.772		**0.019**	1.697	1.091-2.640
CA199	< 35 kU/L							
	≥ 35 kU/L	0.193	1.412	0.840-2.371		0.105	1.509	0.917-2.483
IVE	NO							
	YES	**<0.001**	4.486	2.638-7.631		**<0.001**	4.949	2.996-8.174

### Association between IVE and clinicopathologic characteristics

**Figure 2:**
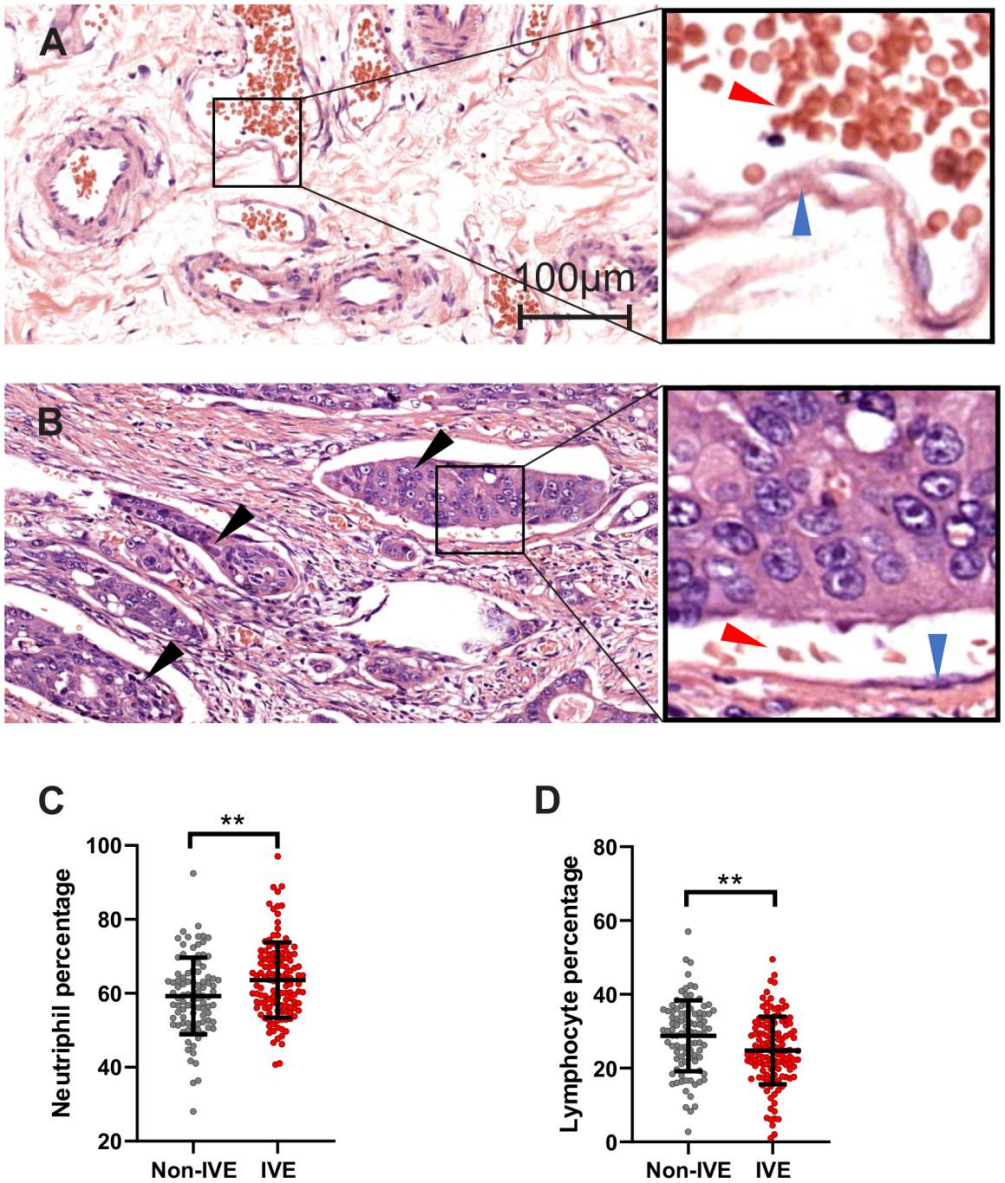
**IVE related to peripheral lymphocyte and neutrophil percentage.** (**A**, **B**) representative H&E staining images of cancer tissue without IVE (**A**) and with IVE (**B**). IVE is indicated by black arrows, the wall of vessels and red cells are indicated by blue arrows and red arrows, respectively. (**C**, **D**) IVE patients had higher neutrophil percentage (**C**) and lower lymphocyte percentage (**D**) relative to non-IVE patients. (**, *P* < 0.01).

In cohort 1, IVE was observed in 126 (57.3%) specimens (Figure 2A, 2B). Our analysis revealed that IVE was associated with lymph node metastasis (*P* < 0.001), tumor histologic type (*P* < 0.001) and pre-operation obstruction symptom (*P* = 0.008) (Supplementary Table 2). Importantly, patients with IVE had a higher neutrophil percentage (non-IVE group, 59.26 ± 10.38 versus IVE group, 63.38 ± 10.17; *P* = 0.002) and lower lymphocyte percentage (non-IVE group, 28.77 ± 9.62 versus IVE group, 24.80 ± 9.19; *P* = 0.002) (Figure 2C, 2D and Supplementary Table 3). Additionally, serum creatinine, a marker for renal function assessment, was higher in the IVE group (*P* = 0.008) (Supplementary Table 3).

### Signatures of differential gene expression between groups

**Figure 3:**
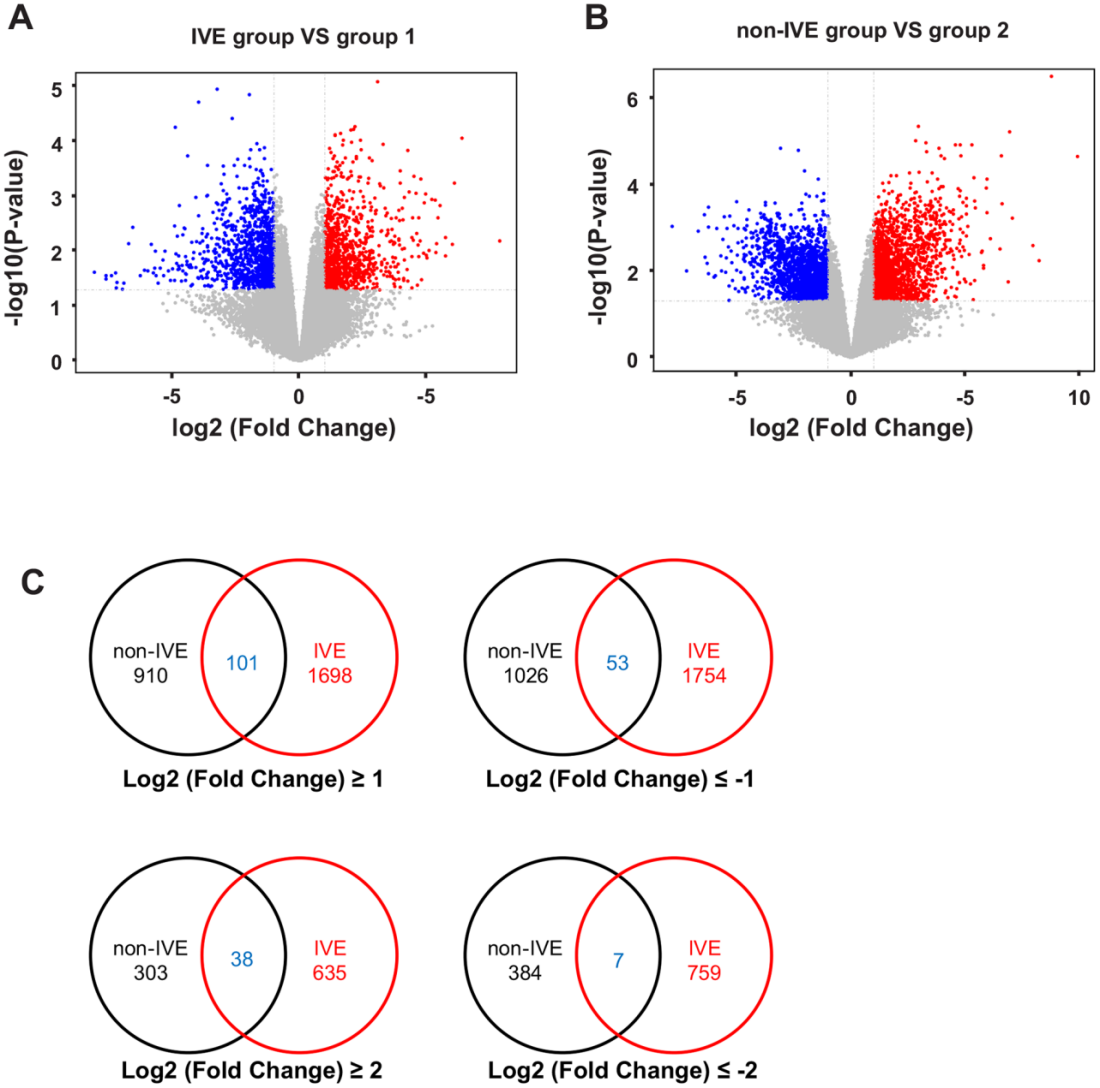
**Analysis of DEGs in non-IVE and IVE groups compared with their paired normal groups.** (**A**) Volcano plot of DEGs between the IVE and paired group 1. (**B**) Volcano plot of DEGs between IVE and paired group 2. For (**A**, **B**), Genes significantly up-regulated are highlighted in red while down-regulated genes are highlighted in blue. The *P*-value threshold and cutoff of absolute fold change were <0.05 and >2, respectively. (**C**) Venn diagrams indicating the number of DEGs in each group and common DEGs. IVE, IVE group versus paired group 1; non-IVE: non-IVE group versus paired group 2.

A total of 12 samples from 6 patients (Supplementary Table 4) with stage III CRC were used for microarray analysis. These samples consisted of 6 fresh cancer tissues and their paired non-cancerous tissues. Compared with paired group 2, there were 1011 up-regulated genes and 1080 down-regulated genes with absolute fold change >2 and *P* < 0.05 in the non-IVE group (Figure 3A). Meanwhile, there were 1799 up-regulated genes and 1807 down-regulated genes with absolute fold change >2 and *P* < 0.05 in the IVE group compared with paired group 1 (Figure 3B). The number of DEGs in the IVE group was higher compared to the non-IVE group, however, only a small number of genes were shared by both groups (Figure 3C).

**Figure 4:**
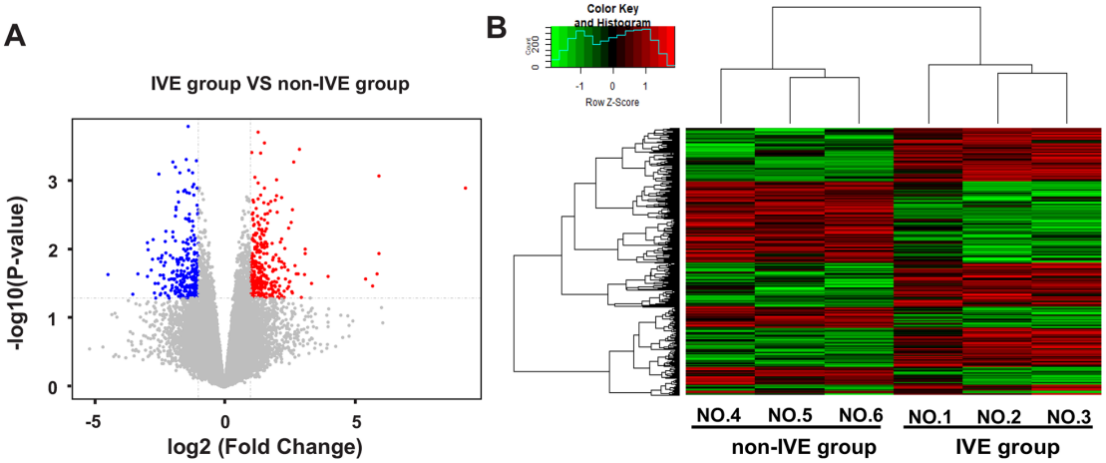
**DEGs and hierarchical clustering analysis in the IVE group compared with the non-IVE group.** (**A**) Volcano plot of DEGs between the IVE group and the non-IVE group. Genes significantly up-regulated are highlighted in red while down-regulated genes are highlighted in blue. The *P*-value threshold and cutoff of absolute fold change were <0.05 and >2, respectively. (**B**) Heat map clustering of gene expression in cancer tissue resected from six patients. Each column represents 1 individual patient tumor and each row represents 1 gene. Color indicate normalized counts of each gene, with red representing higher expression and green relatively lower expression.

Next, the IVE group was compared with the non-IVE group. There were 670 genes with fold change > 2 (306 up-regulated and 264 down-regulated) and 93 genes with fold change > 4 (52 up-regulated and 41 down-regulated) compared with the non-IVE group (Figure 4A). The top ten up- and down-regulated genes were listed in Supplementary Table 5. DEGs clustering analysis was performed and it was revealed that the observed gene signatures distinguished IVE tumors from non-IVE tumors effectively (Figure 4B).

### CRC with IVE had a more immunosuppressive tumor microenvironment (TME)

**Figure 5:**
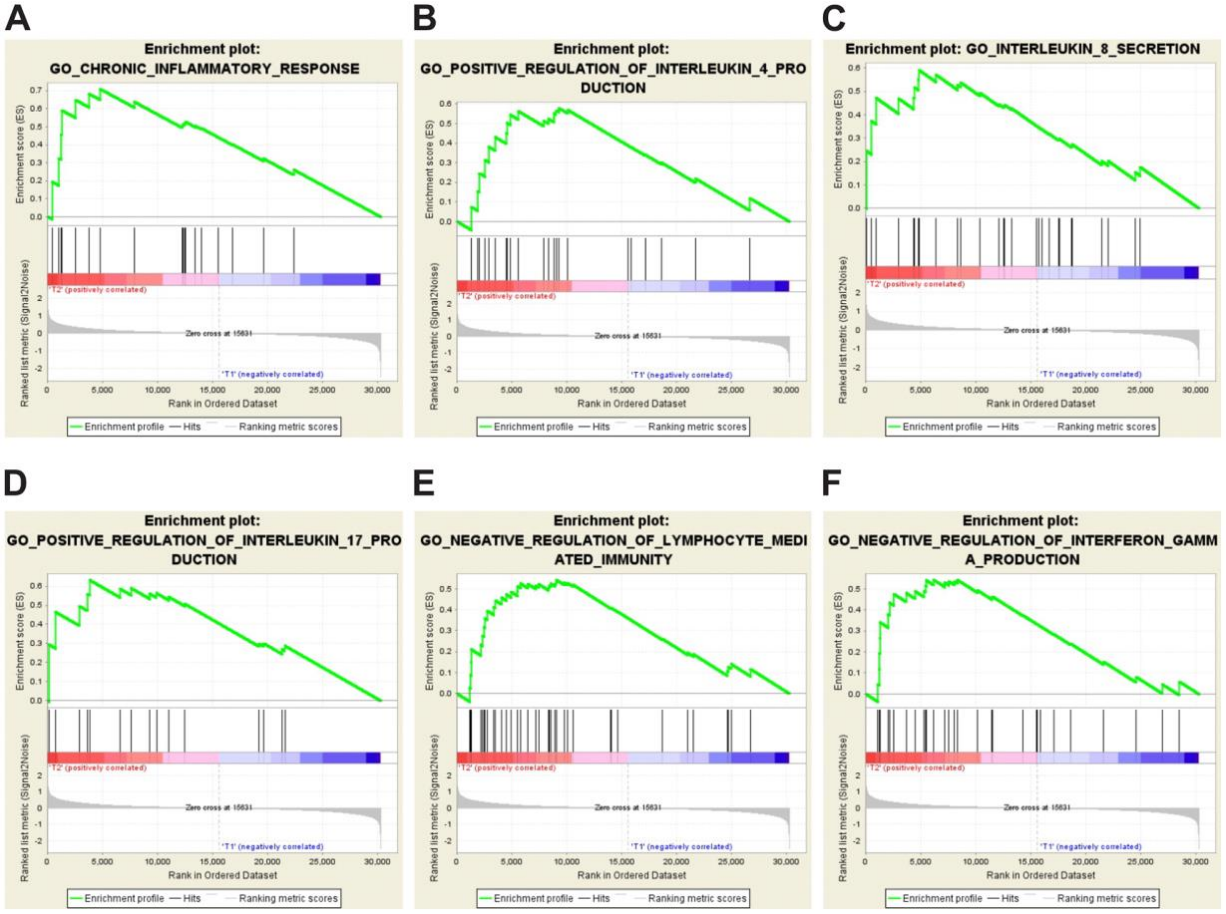
**GSEA-enrichment plots of representative gene sets in the IVE group.** (**A**) chronic inflammatory response (NES,1.777; FDR q-value, 0.012; NOM p-value, <0.001). (**B**) positive regulation of IL-4 production (NES, 1.549; FDR q-value, 0.023; NOM p-value, 0.028). (**C**) IL-8 secretion (NES, 1.654; FDR q-value, 0.034; NOM p-value, 0.006). (**D**) positive regulation of IL-17 production (NES, 1.572; FDR q-value, 0.025; NOM p-value, 0.021). (**E**) negative regulation of lymphocyte mediated immunity (NES, 1.631; FDR q-value, 0.022; NOM p-value, 0.009). (**F**) negative regulation of IFNγ production (NES, 1.561; FDR q-value, 0.024; NOM p-value, 0.031).

The results above (Figure 2 and Supplementary Table 3) suggest that inflammation and immune response may play a role in IVE development. To identify enriched genes that may be involved in immune response or inflammation, GSEA analysis was performed by using gene expression data and some prior defined gene sets. Our analysis revealed that the IVE group correlated with chronic inflammatory response positively and lymphocyte-mediated immunity negatively compared with the non-IVE group (Figure 5). Additionally, cytokine/chemokine production pathways were also analyzed between IVE and non-IVE group. Gene sets promoting the production of some interleukin (IL) including IL-4, IL-8, and IL-17 and negatively regulating interferon γ (IFNγ) production were enriched in the IVE group (Figure 5). Differential enrichment of those gene sets was not observed between the group 1 and group 2. Thus, the chronic inflammatory and immunosuppressive tumor microenvironment may promote IVE formation and CRC progression.

### CRC with IVE had fewer CD3^+^ and CD8^+^ TILs

CD3^+^ and CD8^+^ subsets represent the total infiltrating T cells and cytotoxic T cells, respectively.

**Figure 6:**
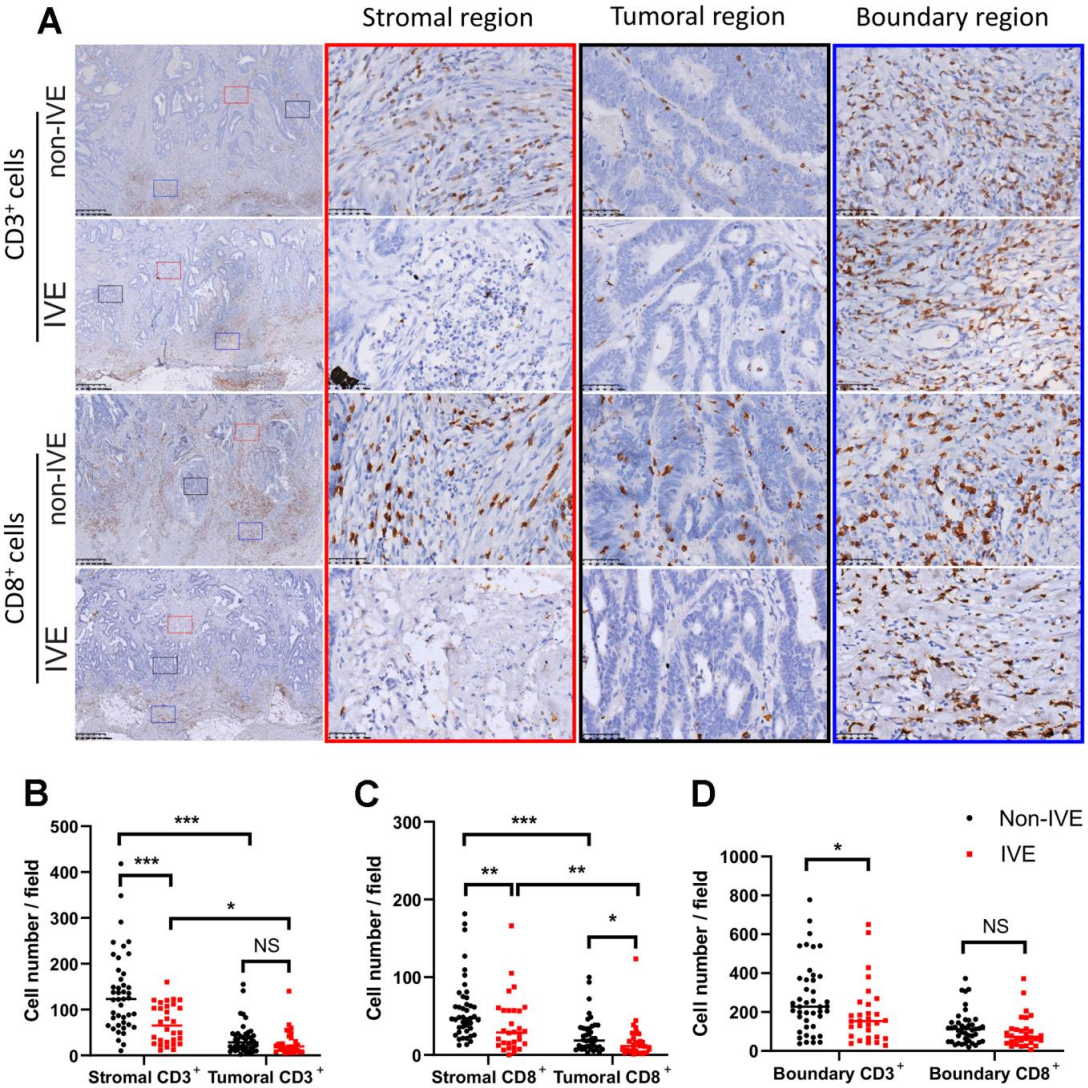
**CRC with IVE had fewer CD3^+^ and CD8^+^ TILs.** (**A**) representative images of CD3^+^ and CD8^+^ TILs in different regions of tumors with or without IVE. Stromal, tumoral, and boundary regions are indicated by red, black and blue rectangles, respectively. (**B**) tumor with IVE had fewer stromal CD3^+^ TILs compared with non-IVE. (**C**) tumors with IVE had fewer stromal and tumoral CD8^+^ TILs compared with non-IVE. (**D**) tumors with IVE had fewer boundary CD3^+^ TILs but similar CD3^+^ TILs. (NS, *P* > 0.05; *, *P* < 0.05; **, *P* < 0.01; ***, *P* < 0.001).

The density of TILs reflects the immune status in TME. We found TILs did not distribute equally in tumor areas including stromal region, tumor region, and boundary region (Figure 6). Compared to tumors without IVE, those with IVE had fewer CD3^+^ and CD8^+^ TILs in both stromal and tumoral regions, while fewer CD8^+^ TILs were detected in tumoral regions (Figure 6A–6C). Notably, TILs were more abundant in the stromal region compared with tumor region (Figure 6A–6C), which suggested a pivotal role for the stroma in immune-mediated cancer control. TILs accumulated prominently at the frontier of the tumor (Figure 6A), however, only a small difference of CD3^+^ TILs was observed between non-IVE and IVE tumors (Figure 6D).

## DISCUSSION

Except for the TNM system, molecular markers like microsatellite instability (MSI), KRAS and BRAF mutations can predict the prognosis and direct the treatment of specific stage CRC [14, 18]. For patients with stage III CRC, the recommended treatment is radical surgery along with local radiotherapy and/or systemic chemotherapy regardless [19]. Though patients receive the same treatment, the outcome varies a lot and is largely unsatisfactory. It is necessary to identify new prognostic markers to help modify the therapeutic regimen for patients with stage III CRC.

According to the AJCC staging system [20], lymph node metastasis is the main characteristic of stage III CRC, which indicates regional spread via lymphatic vessels. But death due to distant organ metastasis via hematogenous spread is common. Identifying the metastatic process in its early stages is critical. Unlike the detection of CTCs or ctDNA, the presence of IVE indicates cluster of tumor cells have survived and will likely develop into distant metastasis. Previous studies found vascular invasion was an unfavorable prognostic factor in various solid cancers including CRC [2, 12, 21, 22]. In our study, over half of stage III CRC patients analyzed presented with IVE and had a worse prognosis, which was consistent with previous findings [3]. It has been established that patients with stage IV CRC can benefit from targeted therapy [23–25]. Given our findings outlines above and previous research, the survival time of stage III CRC patients with IVE is comparable with stage IV CRC patients. It’s a question whether it is appropriate to treat those as local advanced cancer. Further clinic research is needed to find out if those patients can benefit from other treatment such as target therapy.

IVE was observed decades ago [4, 5, 11], but the molecular mechanism is still unclear due to a lack of validated models that mimic the process. Chronic inflammation and immune response are closely related to the development of many cancers including CRC [26, 27]. We previously found some indices related to inflammatory and immune response could serve as prognostic predictors [28]. In this study, CRC with IVE correlated with higher neutrophil and lower lymphocyte percentage. Patients who showed obstruction symptoms also had higher chances of developing IVE. Moreover, the enrichment of gene sets including chronic inflammation pathway and negative regulation of lymphocyte-mediated immunity in IVE was observed. These findings suggest systematic or local inflammatory and immune responses may contribute to CRC metastasis following IVE formation.

The tumor microenvironment, which consists of immune cells, stromal cells, blood vessels, extracellular matrix, chemokines, and cytokines, interacts with tumor cells consistently and promote evolution and immune tolerance of cancerous cells [29, 30]. The tumor microenvironment is heterogeneous [31] and can also contribute to tumor heterogeneity along with genetic divergence and epigenetic regulation [32]. To characterize the tumor microenvironment, a microarray-based analysis was performed in our study. The number of DEGs in cancer tissue with IVE is almost double that in cancer tissue without IVE when compared with their paired noncancerous tissue, and only a very small number of DEGs were shared by both groups. DEGs clustering analysis also confirmed the distinguishing features between the two groups. Here one limitation is the low number of patients which may lead to some bias. Among those top ten up/down regulated genes in IVE group, some genes have been studied in CRC. Those including REG1A, KLK8, KLK12 and MAGEA are up regulated in IVE group, among which REG1A and KLK8 predict poor prognosis in CRC [33, 34], while silence of KLK12 and MAGEA can inhibit CRC growth and promote cell death [35, 36]. Interestingly, cytoplasmic ASPN have been found to promote cell migration and indicate a poor prognosis in colorectal cancer [37], and elevated DKK1 expression is associated with recurrence and impairs the response to PD-1 blockade in dMMR CRC [38], while both of which are down regulated in IVE group. PTF1A has been well studied in pancreatic cancer. It can block pancreatic tumorigenesis and re-differentiate pancreatic cancer precursors to exocrine cells [37]. DRD2 serves as tumor suppressor and triggers pyroptosis in breast cancer, while its overexpression promotes CRC progression [39, 40]. Furth research should be carried out to reveal more functions of those genes.

The survival, proliferation, and metastasis of cancer cells are influenced by many kinds of cytokines interacting with them in the tumor microenvironment. A previous study showed IVE was characterized by positively expressing CD133, a surface marker of stem-like cells [3]. IL-4 has been found to maintain the stemness of CRC and mediate resistance to apoptosis via the autocrine response. Blocking IL-4 can synergize with chemotherapeutic drugs to overcome the resistance of CSCs to apoptosis [41, 42]. Tumor-derived IL-8 can bias the tumor microenvironment towards an immunosuppressive state and promote tumor invasion, metastasis and resistance to chemotherapy. Angiogenesis of endothelial cells upon IL-8 stimulation is necessary for tumor growth by delivering essential nutrients [43]. Similarly, Th17 cells and IL-17 contribute to carcinogenesis, angiogenesis, and tumor resistance to anti-angiogenic therapy and predict poor prognosis of CRC [44–47]. On the contrary, IFNγ has antitumor effects by both acting on tumor cells directly and remodeling the tumor microenvironment, including function modulation of tumor-infiltrating immune cells and stromal cells. Resistance to checkpoint blockade was found to be associated with genomic defects in the IFNγ pathway in tumor cells [48]. Our GSEA results indicated that pathways inducing IL-4, IL-8 and IL-17 production were activated in the IVE group while IFNγ production was inhibited. This may contribute to IVE development, which may ultimately lead to an immunosuppressive tumor microenvironment. TILs with specific CD3 and CD8 markers existing in different compartments of the tumor were examined to evaluate the Immunoscore in CRC tumors [49]. Generally, higher levels of CD3^+^ or CD8^+^ TILs generally correlate with a better prognosis in CRC patients [50, 51]. In this study, we found a significant difference in both CD3^+^ and CD8^+^ TILs related to different regions between IVE and non-IVE tumors, indicating a relatively immunosuppressive tumor microenvironment within an IVE tumor. Additionally, immune heterogeneity was observed within a single tumor for the distribution of TILs was quite different.

## CONCLUSIONS

Stage III CRC patients with IVE have a worse prognosis compared to those without IVE. The tumor microenvironment, which is characterized by chronic inflammation and immunosuppression, may be responsible for IVE development. For both distinct clinical and biological behavior, consideration should be made whether it is appropriate to consider all stage III CRC as regional tumors. As indicated by the findings in this paper, the presence of IVE may suggest tumor metastasis.

## Supplementary Material

Supplementary Figure 1

Supplementary Tables
