# Supplementary material for: Intravascular emboli relates to immunosuppressive tumor microenvironment and predicts prognosis in stage III colorectal cancer
Source: Aging (Albany NY). 2021 Aug 26;13(16):20609–28. doi: 10.18632/aging.203451 (PMC8436899; doi:10.18632/aging.203451)
Supplement: Supplementary Figure 1 [file aging-13-203451-s001.pdf]

SUPPLEMENTARY FIGURE

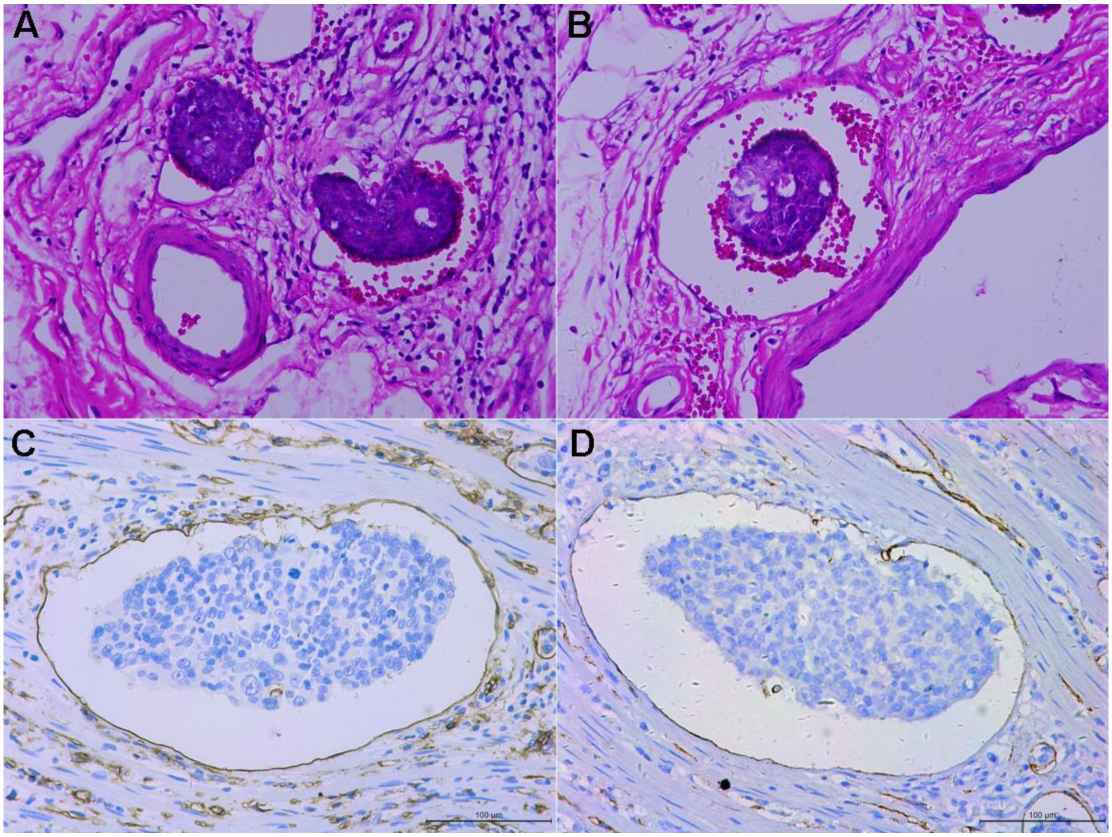

Supplementary Figure 1. Intravascular emboli (IVE) diagnostic criteria by HE and IHC staining. (**A**, **B**) IVE diagnosed by HE staining: a cluster of tumor cells in an endothelium-lined space either surrounded by a rim of smooth muscle or containing red blood cells; (**C**, **D**) IVE diagnosed by IHC staining: blood vessel endothelium strongly stained by CD34 antibody but not stained by D2-40.
