# Supplementary material for: Intravascular emboli relates to immunosuppressive tumor microenvironment and predicts prognosis in stage III colorectal cancer
Source: Aging (Albany NY). 2021 Aug 26;13(16):20609–28. doi: 10.18632/aging.203451 (PMC8436899; doi:10.18632/aging.203451)
Supplement: Supplementary Tables [file aging-13-203451-s002.pdf]

SUPPLEMENTARY TABLES

Supplementary Table 1. Baseline demographic and clinical characteristics of patients.

Parameters	N	Range	Minimum	Maximum	Mean
Age (year)	220	69.00	14.00	83.00	55.710
Total protein (g/dL)	220	35.50	48.10	83.60	66.689
Albumin (g/dL)	220	48.30	4.10	52.40	39.883
Globulin (g/dL)	219	26.40	15.30	41.70	26.646
A/G ration	220	1.40	0.90	2.30	1.539
Total bilirubin (µmol/L)	220	59.60	0.20	59.80	10.855
Direct bilirubin (µmol/L)	220	28.10	0.90	29.00	4.163
ALT (U/L)	220	149.10	3.20	152.30	19.925
AST (U/L)	220	104.30	9.90	114.20	22.488
Urea (mmol/L)	220	13.50	1.16	14.66	4.677
Creatinine (µmol/L)	220	131.80	23.60	155.40	79.018
Glucose (mmol/L)	209	11.76	2.34	14.10	5.263
Triglyceride (mmol/L)	184	9.36	0.37	9.73	1.306
Cholesterol (mmol/L)	184	7.28	1.47	8.75	4.540
HDL (mmol/L)	184	2.12	0.22	2.34	1.266
LDL (mmol/L)	184	22.60	0.00	22.60	2.884
HDL/CHO	184	2.11	0.15	2.26	0.295
Potassium (mmol/L)	220	2.60	2.42	5.02	3.985
Sodium (mmol/L)	220	27.60	121.10	148.70	141.606
Chloride (mmol/L)	220	14.70	95.40	110.10	103.829
Calcium (mmol/L)	220	0.88	1.69	2.57	2.254
WBC count (10^3/mL)	220	13.20	2.40	15.60	6.228
RBC count (10^6/mL)	220	3.10	2.61	5.71	4.083
Hemoglobin (g/L)	220	149.00	16.00	165.00	117.370
Platelet count (10^3/mL)	220	681.00	21.00	702.00	230.250
Neutrophil count (10^3/mL)	220	12.60	0.90	13.50	3.961
Lymphocyte count (10^3/mL)	220	3.80	0.10	3.90	1.573
Monocyte count (10^3/mL)	220	1.30	0.10	1.40	0.486
Neutrophil percentage (%)	220	69.00	28.00	97.00	61.733
Lymphocyte percentage (%)	220	56.00	1.00	57.00	26.495
Monocyte percentage (%)	220	17.20	0.70	17.90	8.070
Thrombocytocrit (%)	220	0.50	0.00	0.50	0.202
MPV (fl)	220	9.70	6.20	15.90	8.822
PDW (%)	220	15.50	7.00	22.50	16.799
PT (second)	220	13.90	10.20	24.10	12.729
INR	220	1.15	0.79	1.94	0.982
APTT (second)	220	28.20	23.20	51.40	34.376
TT (second)	220	63.60	13.90	77.50	17.726
Fibrinogen (g/L)	219	6.27	1.61	7.88	3.663

Abbreviations: A/G, albumin to globulin ration; ALT, Alanine aminotransferase; AST, Aspartate aminotransferase; HDL, high-density lipoprotein; LDL, low-density lipoprotein; HDL/CHO, high-density lipoprotein to cholesterol ration; WBC, white blood cell; RBC, red blood cell; MPV, mean platelet volume; PDW, platelet distribution width; PT, prothrombin time; INR, international normalized ratio; APTT, activated partial thromboplastin time; TT, thrombin time.

Supplementary Table 2. Relationships between clinical characteristics and IVE.

Parameters	non-IVE group	IVE group	*P*-value
Age			
≤60	55	77	0.697
>60	39	49	
Gender			
Male	47	79	0.060
Female	47	47	
Location			
Left colon	18	23	0.241
Right colon	23	20	
Rectum	53	83	
Invasion depth			
T1-2	14	9	0.063
T3-4	80	117	
Lymph node metastasis			
pN1	65	55	**<0.001**
pN2	29	71	
Histologic type			
Mucinous/mix	14	15	**<0.001**
Poor	6	43	
Well /Moderate	74	68	
Tumor Morphology			
Ulcerative	44	57	0.472
Infiltrating	3	8	
Protruded	46	53	
Diameter			
<5cm	45	73	0.070
≥5cm	48	47	
Obstruction symptoms			
No	93	113	**0.008**
Yes	1	12	
CEA (ng/ml)			
<5	64	83	0.095
≥5	28	37	
CA199 (kU/L)			
<35	77	96	0.491
≥35	15	24	
CA242 (kU/L)			
<20	73	95	0.806
≥20	16	19	
Adjuvant chemotherapy			
FOLFOX4/6	58	78	0.737
XELOX	7	10	
Other	2	6	
No chemotherapy	27	32	

Supplementary Table 3. Difference of blood routine and chemistry between groups.

Parameters	non-IVE group	IVE group	*P*-value
Total protein (g/dL)	66.38±6.12	66.92±6.51	0.539
Albumin (g/dL)	39.92±4.39	39.86±5.34	0.929
Globulin (g/dL)	26.47±4.26	26.78±4.45	0.600
A/G ration	1.54±0.27	1.53±0.29	0.783
Total bilirubin (µmol/L)	10.98±7.40	10.76±6.01	0.812
Direct bilirubin (µmol/L)	4.11±3.31	4.20±2.47	0.822
ALT (U/L)	21.2±15.82	19.1±17.07	0.396
AST (U/L)	22.51±9.51	22.47±13.66	0.980
Urea (mmol/L)	4.58±1.62	4.75±1.81	0.474
Creatinine (µmol/L)	74.7±19.2	82.24±20.34	**0.006**
Glucose (mmol/L)	5.17±1.08	5.34±1.34	0.330
Triglyceride (mmol/L)	1.29±1.08	1.32±1.17	0.828
Cholesterol (mmol/L)	4.53±1.02	4.54±0.99	0.940
HDL (mmol/L)	1.24±0.26	1.29±0.32	0.249
LDL (mmol/L)	2.77±0.96	2.97±2.12	0.424
HDL/CHO	0.28±0.07	0.31±0.20	0.309
Potassium (mmol/L)	3.94±0.43	4.02±0.38	0.156
Sodium (mmol/L)	141.58±3.48	141.63±2.73	0.909
Chloride (mmol/L)	104.29±2.82	103.49±3.10	0.050
Calcium (mmol/L)	2.25±0.13	2.25±0.12	0.968
WBC count (10^3/mL)	6.01±2.05	6.39±2.29	0.213
RBC count (10^6/mL)	4.04±0.58	4.11±0.58	0.344
Hemoglobin (g/L)	116.12±23.26	118.31±24.56	0.504
Platelet count (10^3/mL)	225.67±94.72	233.67±95.28	0.537
Neutrophil count (10^3/mL)	3.68±1.82	4.17±2.04	0.066
Lymphocyte count (10^3/mL)	1.66±0.59	1.51±0.65	0.093
Monocyte count (10^3/mL)	0.48±0.18	0.49±0.20	0.665
Neutrophil percentage (%)	59.26±10.38	63.38±10.17	**0.002**
Lymphocyte percentage (%)	28.77±9.62	24.80±9.19	**0.002**
Monocyte percentage (%)	8.23±2.81	7.95±2.82	0.480
Thrombocytocrit (%)	0.20±0.08	0.20±0.08	0.590
MPV (fl)	8.95±1.69	8.73±1.33	0.268
PDW (%)	16.77±1.43	16.82±0.75	0.727
PT (second)	12.71±0.88	12.74±1.40	0.839
INR	0.98±0.08	0.99±0.12	0.520
APTT (second)	34.58±4.56	34.22±5.02	0.583
TT (second)	17.19±1.41	18.1±5.61	0.116
Fibrinogen (g/L)	3.74±1.06	3.60±0.80	0.289

Abbreviations: A/G, albumin to globulin ration; ALT, Alanine aminotransferase; AST, Aspartate aminotransferase; HDL, high-density lipoprotein; LDL, low-density lipoprotein; HDL/CHO, high-density lipoprotein to cholesterol ration; WBC, white blood cell; RBC, red blood cell; MPV, mean platelet volume; PDW, platelet distribution width; PT, prothrombin time; INR, international normalized ratio; APTT, activated partial thromboplastin time; TT, thrombin time.

Supplementary Table 4. Six patients for microarray analysis.

Patients	IVE	Age	Gender	TNM stage	LY%	NE%	Location	Lymph node metastasis	Histologic type
NO.1	YES	61	Male	T4aN1bM0	27.5	33.0	Rectum	2/13	Moderate
NO.2	YES	60	Male	T4aN1bM0	24.0	66.7	Sigmoid	3/11	Moderate
NO.3	YES	62	Male	T4aN2bM0	16.8	77.9	Rectum	7/12	Moderate
NO.4	NO	52	Female	T4aN1bM0	32.0	57.0	Rectum	3/18	Moderate
NO.5	NO	43	Male	T4aN2aM0	37.2	57.3	Rectum	6/11	Moderate
NO.6	NO	63	Male	T4aN1aM0	41.0	42.7	Rectum	2/14	Moderate

Supplementary Table 5. Top ten up/down regulated genes in IVE group.

Genes	log2 (Fold change)	*P*-value
CALB1	9.250	0.001
REG1A	5.930	0.011
DSG3	5.928	0.001
KLK8	5.866	0.022
ABHD12B	5.697	0.034
XLOC_004031	5.419	0.026
KLK12	3.993	0.024
MAGEA6	3.355	0.031
KRT16P2	3.105	0.011
PLA2G4D	3.104	0.010
XLOC_011804	-2.677	0.042
RIIAD1	-2.731	0.007
ASPN	-2.748	0.014
XLOC_l2_015529	-2.801	0.020
DRD2	-2.935	0.010
ZBTB16	-2.943	0.024
XLOC_012248	-2.945	0.008
XLOC_l2_010573	-3.307	0.023
PTF1A	-3.526	0.044
DKK1	-4.461	0.023
